# A toolkit for incorporating genetics into mainstream medical services: Learning from service development pilots in England

**DOI:** 10.1186/1472-6963-10-125

**Published:** 2010-05-14

**Authors:** Catherine L Bennett, Sarah E Burke, Hilary Burton, Peter A Farndon

**Affiliations:** 1 NHS National Genetics Education and Development Centre, Birmingham Women's NHS Foundation Trust, Edgbaston, Birmingham, B15 2TG, UK; 2 School of Education, University of Birmingham, Edgbaston, Birmingham, B15 2TT, UK; 3 PHG Foundation, Strangeways Research Laboratory, Worts Causeway, Cambridge, CB1 8RN, UK

## Abstract

**Background:**

As advances in genetics are becoming increasingly relevant to mainstream healthcare, a major challenge is to ensure that these are integrated appropriately into mainstream medical services. In 2003, the Department of Health for England announced the availability of start-up funding for ten 'Mainstreaming Genetics' pilot services to develop models to achieve this.

**Methods:**

Multiple methods were used to explore the pilots' experiences of incorporating genetics which might inform the development of new services in the future. A workshop with project staff, an email questionnaire, interviews and a thematic analysis of pilot final reports were carried out.

**Results:**

Seven themes relating to the integration of genetics into mainstream medical services were identified: planning services to incorporate genetics; the involvement of genetics departments; the establishment of roles incorporating genetic activities; identifying and involving stakeholders; the challenges of working across specialty boundaries; working with multiple healthcare organisations; and the importance of cultural awareness of genetic conditions.

Pilots found that the planning phase often included the need to raise awareness of genetic conditions and services and that early consideration of organisational issues such as clinic location was essential. The formal involvement of genetics departments was crucial to success; benefits included provision of clinical and educational support for staff in new roles. Recruitment and retention for new roles outside usual career pathways sometimes proved difficult. Differences in specialties' working practices and working with multiple healthcare organisations also brought challenges such as the 'genetic approach' of working with families, incompatible record systems and different approaches to health professionals' autonomous practice.

'Practice points' have been collated into a Toolkit which includes resources from the pilots, including job descriptions and clinical tools. These can be customised for reuse by other services.

**Conclusions:**

Healthcare services need to translate advances in genetics into benefits for patients. Consideration of the issues presented here when incorporating genetics into mainstream medical services will help ensure that new service developments build on the body of experience gained by the pilots, to provide high quality services for patients with or at risk of genetic conditions.

## Background

Genetics is increasingly widely relevant to mainstream healthcare [1,2]. Patients with or at risk of heritable conditions are cared for across health services and advances in the prevention, diagnosis, treatment and management of single gene disorders, as well as in our understanding of the genetic component and familial implications of common diseases, mean that services for patients may be enhanced by integrating genetic activities into clinical practice [3-7].

In the UK, specialist genetics services are organised regionally and provided by Consultant Clinical Geneticists and Genetic Counsellors, in close collaboration with specialist laboratories, in Regional Genetics Centres (RGCs) [8]. However, as genetics becomes increasingly relevant within an area of practice, new 'genetic' services need to be developed and integrated. For example, as our understanding of the role of inheritance in certain types of cancer has increased, cancer family history clinics have been established [9].

Through its 2003 Genetics White Paper, "Our inheritance, our future - realising the potential of genetics in the NHS [National Health Service]" [10], the Department of Health for England (DH) provided two years start-up funding for ten pilot service development projects, which they termed 'Mainstreaming Genetics'. These pilots were established to explore the various aspects of this genetic integration that would be important for overall success and to develop innovative service models.

In establishing new services, the pilots developed new roles, new patient pathways and new ways of working. They gained considerable experience of how to integrate genetics into mainstream healthcare services. The NHS National Genetics Education and Development Centre [11], also established through the Genetics White Paper, was given the remit to support the pilots and to disseminate their experiences of integrating genetic activities into other mainstream medical services to the wider NHS. The DH also funded an external evaluation team who worked with the pilots to explore the effectiveness of the service developments [12].

This paper explores the challenges faced by the pilots, the factors that contributed to the successful integration of genetic activities into mainstream medicine and the barriers they faced establishing new roles and services so that those developing future services can build on this experience.

## Methods

**Figure 1:**
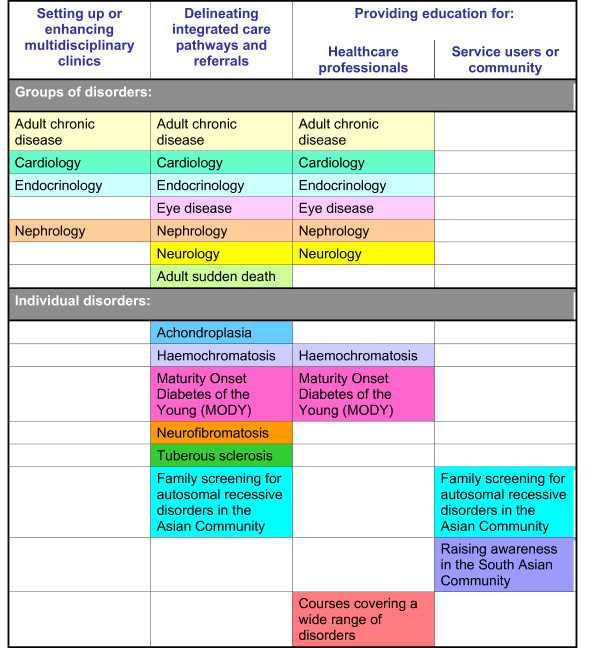
**Overview of pilot services: main themes**.

The ten 'Mainstreaming Genetics' service development pilots covered a range of genetic conditions, clinical specialities and service models, including: setting up or enhancing multidisciplinary clinics; delineating integrated care pathways and referrals; and providing education for either other health professionals or service users and the community (Figure 1). Pilot services are listed in Table 1. Multiple methods were used to collate the key experiences of the pilots that might inform future service developments. Advice from the Central Office for Research Ethics Committees (COREC) indicated that ethical committee approval was not required for this service development work.

**Table 1:** Pilot services and new clinical roles

*Pilot service*	*New clinical roles:*	
	
	Genetic Counsellor working mainly outside the specialist genetics service	Nurse specialist in a mainstream specialty undertaking work in genetics in that specialty
The integration and development of renal genetics and nephrology services	(No information)	(No information)
Developing mainstream genetic services in renal, cardiac and endocrine genetics	-	Nurse specialist in renal genetics Nurse specialist in cardiac genetics Nurse specialist in endocrine genetics
Community based, hospital linked genetic services for extended family members of consanguineous Asian families affected by autosomal recessive genetic disorders	Specialist genetic health visitor	-
DialGEN - Developing a Liaison Service for Genetics in Medicine	DialGEN genetic counsellor	Arrhythmia nurse specialist - genetics liaison High risk midwife - genetics liaison Haemophilia nurse specialist - genetics liaison
Delivering genetics within the community	Community genetic counsellor	-
Genetic educational seminars. A targeted education programme for health professionals covering a wide range of genetic diseases	(No information)	(No information)
'Genetics in Health' - a community project	(No information)	(No information)
Developing integrated care pathways and guidelines for routine monitoring of selected genetic disorders in primary and secondary care	-	Senior cardiac liaison nurse
Bringing genetics into mainstream clinical practice: Haemochromatosis	-	Extended role for liver/alcohol nurse specialist in gastroenterology service
Development of specialist ophthalmic-genetic counsellor and care pathways	Ophthalmic genetic counsellor	-

### 1. Workshop with pilot service staff

As the initial two year funding period was ending, representatives from all the pilot services were invited to attend a one-day workshop to: describe the new roles developed for their services; outline the genetic activities and competences required for their service; discuss their experiences of what was involved in establishing new genetics services in mainstream medicine; share information on any resources used or developed; and explore any gaps in support for their service developments.

### 2. Email questionnaire and interviews

Key points raised at the workshop were used to inform the development of a questionnaire which was sent by email to all ten pilots approximately one month after the workshop. The questionnaire asked about: their experiences of developing substantive new roles; implications of service developments for genetics departments; important factors for developing successful services; barriers to success; formal learning and resources developed by the new service or used to support the pilot; and gaps in provision of support. The questionnaire used a combination of 5-point Likert type responses (1 = low to 5 = high) and free text boxes. Where pilots were unable to complete the questionnaire, a face to face interview covering these issues was offered as an alternative.

### 3. Analysis of final reports

Each pilot produced a final report for the DH and some of these were made available to the team. A thematic analysis of those final reports that were available was carried out. One member of the team generated an initial list of themes which was then discussed within the team and amended until agreement was reached.

Data from these different sources were triangulated to identify key issues that should be considered when planning service developments for genetics in mainstream medicine.

## Results

There were ten pilot service developments. Eight people representing seven of the pilots attended the workshop. Seven pilots returned questionnaires and staff from two others were interviewed. Six final reports were available for analysis. All pilots except one provided two or more sources of information. From the range of data sources, seven key themes emerged relating to the integration of genetic services into mainstream healthcare. These are explored in turn below.

### Planning to incorporate genetics into mainstream services

The pilots identified a number of challenges in planning services to integrate genetics, including: demonstrating the need for new services; timescales and lead-in time; organisational issues such as clinic space; staff skills and awareness of genetics.

Assessing and demonstrating the need to incorporate genetics into mainstream medical specialties was identified as a major issue by the pilots as these were new services, breaking new ground and often crossing traditional professional and specialty boundaries. Patient satisfaction questionnaires, focus groups, feedback from education sessions, review of the published literature, retrospective audits of practice and reviewing referral data were all reported methods which provided evidence to support the need for new co-ordinated or integrated services for patients.

The 'Mainstreaming Genetics' pilots were awarded start-up funding for two years. As shown in Table 2, a short project timescale was rated as the most important barrier to the success of pilots. One pilot commented:

"*Two years is not enough to successfully develop and implement strategies. As this project required working with the community, it took some time to build and gain trust*." (Questionnaire respondent)

**Table 2:** Barriers to success of services

Main barriers to success	Number of responses (1 = low importance to 5 = high importance):				
	1	2	3	4	5
Short timescale of project	-	-	1	2	4

Low level of knowledge/understanding of genetics in mainstream services/primary care	1	-	1	1	4

Disruption from changing staff	2	-	1	1	3

The report from another pilot highlights the need for adequate lead-in time for services to be established, due to the time required for activities such as making contacts, setting up meetings, promoting the service and collecting baseline data:

"*The lead in time for the project was considerable. The first 12 months were spent collecting the baseline data, mapping the PCT [Primary Care Trust] and promoting the project. It was challenging and time consuming to get our 'foot in the door'*." (Report)

Pilot services also identified the need to address organisation issues, such as investigation of clinic and office space availability, early in the planning stage. Lack of clinic space or basic office facilities such as a desk, computer and telephone were very disruptive to service provision. Some pilots expressed concern that holding clinics in an area of the hospital dedicated to other treatments may be worrying for patients. For example, one pilot report reflects:

"*The clinic is held in the endoscopy centre and some may worry that they are coming for a procedure*." (Report)

This pilot service addressed the issue by developing a letter explaining what the person could expect from the appointment.

As pilot services engaged healthcare professionals from other specialties, some found it was important to assess their core genetic skills and the need for support tools such as referral pathways, guidelines, trigger lists and protocols, during the planning phase. Pilots stressed the importance of drawing on existing resources and expertise, including, for example, adapting existing documentation and drawing on existing expertise within the organisation (for example, for the development of websites and patient information).

Pilots found a low level of awareness of genetics services and of the application of genetics to clinical practice in mainstream specialties and primary care (Table 2). This had implications for disseminating information about the new services. Developing plans for publicising the service to relevant health professionals, through training sessions, targeted mailings or other publicity, was considered key:

"*The success of the project is dependent upon creating and maintaining a high profile of the project amongst all healthcare professionals working in the PCT*." (Report)

Successful reported strategies for improving awareness included establishing and using key contacts in relevant stakeholder groups and organisations, and drawing on existing local meetings, networks of practitioners and websites to disseminate information. Communicating information using clear language and not assuming prior knowledge were considered important. Establishing a name for the service and developing standardised proformas and service information helped to develop a recognisable service identity. Publicising an identified point of contact for queries by health professionals or patients at an early stage of the project was also recommended. One project noted:

"*A centralised service and a point of contact are considered to be an important part of the service... GPs [General Practitioners] have been provided with a point of contact and often telephone to ask about the appropriateness of their referral*." (Report)

Where information about a service was disseminated online, pilot services suggested provision of information within, or linked from, a website already used by the target audience, such as the local PCT, hospital or genetics department websites.

### The involvement of genetics departments

The pilot services stressed the importance of the formal involvement of their Regional Genetics Centre at an early stage in the development of new roles and services delivered in other specialties. Identified benefits included the provision of clinical and educational support for staff in new roles, for example, opportunities to attend clinical meetings, supervision and assessment of clinical work, input into the development of information resources, and providing education sessions for service staff and other health professionals.

As shown in Table 3, establishing a formal commitment to provision of time and named support from the genetics department were considered important. One questionnaire respondent noted:

*"New role practitioners need to have access to experienced mentors for support and information*." (Questionnaire respondent)

**Table 3:** Involvement of specialist genetics department

Main factors for success	Number of responses (1 = low importance to 5 = high importance):				
	1	2	3	4	5
A formal time commitment from the genetics department	-	-	1	-	7
Named support for the new roles/practitioners	-	-	1	-	7
Formal commitment of genetics department to provision of education to practitioners	-	-	1	-	6
Formal commitment of genetics department to assessment of competence	-	-	1	-	6
Formal provision of opportunity to discuss difficult cases	-	-	1	1	5
Formal inclusion of practitioner as part of a designated 'team'	-	-	1	2	5
Formal supervision of clinical work	-	-	-	4	3
Clear designation of one manager for the new practitioner	-	1	1	-	6

Another questionnaire respondent described the line management provided by the genetics department as "invaluable", and a third wrote:

"*Excellent support provided by the manager of the clinical genetics department and the team as a whole in teaching and support to a complete novice to the genetics service and genetic conditions*." (Questionnaire respondent)

A formal commitment from the genetics department to supervision of clinical work, provision of education to practitioners and to assessment of competence were also rated as highly important. One respondent noted:

"*Important because genetics is new, specialist knowledge is concentrated in regional genetic services. They are in the best position to be the 'educators'*." (Questionnaire respondent)

Open comments indicated that formal supervision of clinical work was necessary to ensure safe and up-to-date practice and to assess clinical competencies, one person writing:

"*Theory is great but being able to practice in your own setting is very different*." (Questionnaire respondent)

Another pilot described supported development of the new role, with structured transition from observation of genetic counselling sessions, to supervised practice for six months, before formal assessment and then independent practice.

The opportunity to discuss difficult cases and inclusion within a designated team were also highly rated (Table 3). One person described inclusion in a team as "*an invaluable avenue of support and encouragement*" (questionnaire respondent), whilst another acknowledged the importance of such support for patient safety:

"*Unfamiliar situations mean that being able to consult formally is essential to avoid adverse events and ensure high clinical standards*." (Questionnaire respondent)

### The establishment of roles incorporating genetic activities

The pilots identified a number of issues focussing on the development of roles integrating genetic activities, recruitment and retention of staff and their training.

Table 1 shows the substantive new clinical roles developed for each pilot. These either involved a nurse specialist in a mainstream specialty undertaking work in genetics in that specialty, or, in some cases, a genetic counsellor (GC) developing additional skills and working mainly outside the specialist genetics service. In some cases an additional GC role was created to co-ordinate or provide education for the specialist nurses. Where the focus of the service was on additional work in the community, this usually involved development of a GC role.

As these new clinical roles spanned different specialties and required additional skills and knowledge to traditional roles, competences had to be identified and substantial training and support given to staff. For example, a nurse specialist in a mainstream specialty would need to acquire knowledge and skills in genetics, or a GC would need to develop particular knowledge in another specialty. This work was developmental: there were no pre-existing training routes or accreditation for such education, as highlighted by one pilot service:

"*Because they were such brand new roles, and the roles adapted and changed over time, difficult for us to think about supporting their development... Now we would provide a more structured, formal training programme*." (Questionnaire respondent)

As shown in Table 4, recruitment was rated as an important factor for success of services. Some pilot services reported difficulties recruiting to roles outside what were considered to be the usual career progression or professional development routes, as well as to part-time or fixed term posts:

*"Vital to get the right people. But recruitment for short term contracts is problematic" *(Questionnaire respondent)

*"By the time staff are trained up, within an 18 month contract with no job security beyond that, so they start looking for other jobs 9 months before the end of the contract" *(Questionnaire respondent)

**Table 4:** Factors for success of services

Main factors for success	Number of responses (1 = low importance to 5 = high importance):				
	1	2	3	4	5
Access to data across other departments (e.g. family records)	-	-	-	-	7
Engaging individuals at senior level in organisations at an early stage	-	1	-	-	7
Understanding how other partner organisations work	-	-	1	1	5
Using established networks	-	-	-	3	4
Access to training/support	-	-	1	2	5
Encouraging groups/individuals to participate in developments	-	-	1	2	4
Recruitment	-	-	2	1	4
Career development	1	-	1	2	4

Strategies adopted by pilots included secondments and sharing posts with other services.

Staff retention was also important: as shown in Table 2, disruption from changing staff was an important barrier to success for some pilot services. Open comments revealed that staff changes and sick leave caused major disruption to service provision, especially in new roles where extensive initial training was required. In new and innovative roles, where there is no clear career pathway, perceived lack of opportunities for professional development and promotion may have an impact on staff retention. Career development and access to training and support were both rated important by pilot services (Table 4), who also highlighted the importance of identifying training needs, providing protected time and funding for training, providing opportunities for staff to gain formal qualifications or accreditation and involving regional genetics centre staff in training and development.

### The identification and involvement of stakeholders

The pilots suggested that it was particularly important to secure the involvement of relevant stakeholders when planning and designing services. They explored different approaches to stakeholder involvement.

As shown in Table 4, engaging individuals at senior levels in organisations, such as Chief Executives and Boards, at an early stage and encouraging groups or individuals to participate in developments were considered essential factors for success. This often included demonstrating the relevance and importance of genetics to mainstream services. One pilot service stressed:

"*Ensure as much involvement and generate enthusiasm and support from line and senior managers, particularly at board level*." (Report)

It was also important to engage healthcare professionals in order to raise awareness of the new services, encourage buy-in and ensure knowledge of how to access services. For some services, engagement with patients and the public was important in order to improve equity of access to services from particular groups, such as minority ethnic groups who have previously been under-represented as genetics service users.

Pilots reported that stakeholder involvement could be difficult to achieve, one questionnaire respondent describing it as: "*A one-person mission at times*". Identifying relevant stakeholders was seen as an important step, and for the pilots these included patients, community members, senior managers, clinical personnel, representatives of relevant organisations and representatives of educational groups. Understanding how partner organisations work and using established networks to make contact were recommended by pilot services (Table 4). Methods of stakeholder involvement included face-to-face meetings, questionnaires and focus groups. Stakeholders were involved in planning and design of services and service documents, and in providing feedback on leaflets, website content, clinical protocols and tools.

### The challenge of working across specialty boundaries

The pilot services were established on the principle that patients benefit from co-ordinated care, and working across specialties can provide a more streamlined and holistic service. Many of the pilots involved a collaboration between genetics and another specialty. One pilot service highlighted the benefits:

"*Combining all the specialties together into one pathway... has been well received by the families referred to the service*." (Report)

However, different specialties have different cultures and working practices. There may be differences in professional culture, for example, in approaches to multi-disciplinary working and the roles of non-medical professionals. Many of the pilot service developments involved new roles for nurses or GCs, working autonomously within multi-professional teams; such roles may be unusual in some medical specialties. As one pilot report noted:

"*There is a culture difference between clinical genetics which is very multi-disciplinary in which the skills and role of non-medical staff, particularly genetic counsellors, is highly valued and supported. This is in contrast with medical specialties where professions allied to medicine may not lead the team and their role may not be initially recognised or supported*." (Report)

In addition, pilots highlighted that genetics services involve working with families and the familial implications of disorders, rather than focusing on an individual patient, and this is not the usual practice of other specialties:

"*The approach of genetics, which is highly tailored to the needs of the individual and family, does not sit well in a process driven specialty which is highly geared to trafficking patients or their presenting problems through the system. Single system or organ specific medical specialties may focus on the acute problem and are often not geared to dealing with multiple family members and multiple concurrent and newly occurring issues*." (Report)

The report from this pilot service development advised other services to devote time and energy to understanding the existing culture of the specialty and of the practitioners' professions.

Awareness of the level of genetics knowledge within different medical specialties was also considered important. As shown in Table 2, pilot services rated low level of knowledge or understanding of genetics in mainstream services and primary care as one of the main barriers to success. One questionnaire respondent described baseline knowledge of genetics amongst healthcare professionals as "very low", and one report noted:

"*It was found that many of the health professionals were not aware of the referral route, the types of referrals that the genetic service received and where the genetic service was based*." (Report)

This issue is reinforced in a pilot service report, which states:

"*The main barriers to mainstreaming continue to be the lack of appreciation of the genetic contribution to the management of complex multi-system disorders in non-genetic specialties*." (Report)

Communicating information about genetic services using clear language and not assuming prior knowledge were therefore vital. Recommendations for the provision of education to health professionals to support genetics services included matching training to the learning needs and interests of the learners, demonstrating the relevance of genetics to the participants' practice and integrating sessions into existing educational networks.

Another issue arising from working across specialties is the impact of increased workload caused by the introduction of additional activities into an established role. This can be problematic, particularly as a new service may not be a priority for staff working predominantly in other roles and to other targets. One report explains:

"*The current NHS is highly managed, target driven with a negative atmosphere towards change and there is strong resistance towards anything that might affect prescribed targets*." (Report)

Pilot services reported that face-to-face explanation of new services or roles can promote understanding and uptake, and two pilot reports describe how services developed from existing informal relationships between interested clinicians in different specialties. The assignment of one specific line manager for each practitioner was also considered important (Table 3) as working with different specialties and Trusts could lead to involvement of multiple managers and to potential conflicts.

### The challenge of working with multiple healthcare organisations

The pilot services often involved working with multiple healthcare organisations. Their experiences highlighted the importance of considering the differences between these organisations and of identifying relevant national and local policies and guidelines at an early stage. National requirements might include National Institute for Health and Clinical Excellence (NICE) guidelines for particular conditions such as familial breast cancer [13] and familial hypercholesterolaemia [14], relevant National Service Frameworks, and the UK Genetics Testing Network (UKGTN) for genetic testing [15]. Local policies and guidelines will exist for a range of developments, such as patient information, websites, protocols and approvals for setting up clinics, honorary contracts and research governance.

The existence of different policies, guidelines, protocols and systems within different organisations proved problematic for some pilot services which operated across specialties and across Trusts. For example, services had to accommodate different systems for clinical notes and medical records, consent procedures, ordering investigations, testing and reporting procedures, and patient registration. Different electronic record systems were sometimes unable to communicate, resulting in multiple copies of records and duplication of work. Table 4 shows that all seven respondents indicated that access to data across other departments, such as family records, was of high importance to the success of new services.

### The importance of cultural awareness of genetic conditions

Some of the pilots were concerned with raising awareness of genetic conditions and genetic services within local communities in order to improve access to services.

One pilot highlighted that different communities have different knowledge and awareness of genetics and of genetic conditions, stating:

"*Participants frequently said that the lack of understanding behind the concept of genetics could explain why the ... community do not come forward to get genetic advice*." (Report)

A culture of stigma or secrecy surrounding genetic conditions within the community meant that some people were reluctant to seek advice from the genetics service, or were unwilling to talk about genetics with others, even family members. The project concluded that the views of service users are therefore important in establishing how services can be made acceptable and accessible for community members:

"*It is important to explore the range of beliefs and attitudes within the ... community about genetics and the genetic service*." (Report)

The report also highlights the influence of community and religious leaders, for example, on beliefs about inheritance, which can influence whether people access genetic services. Approaches to improving genetic literacy within communities developed by the pilots included: employing a genetic link worker to work in a specific community; using bilingual information resources; using the media, such as community radio shows and community newsletters; working with influential members of the community; and distributing information at community events.

### Disseminating experience: Developing a Toolkit

**Figure 2:**
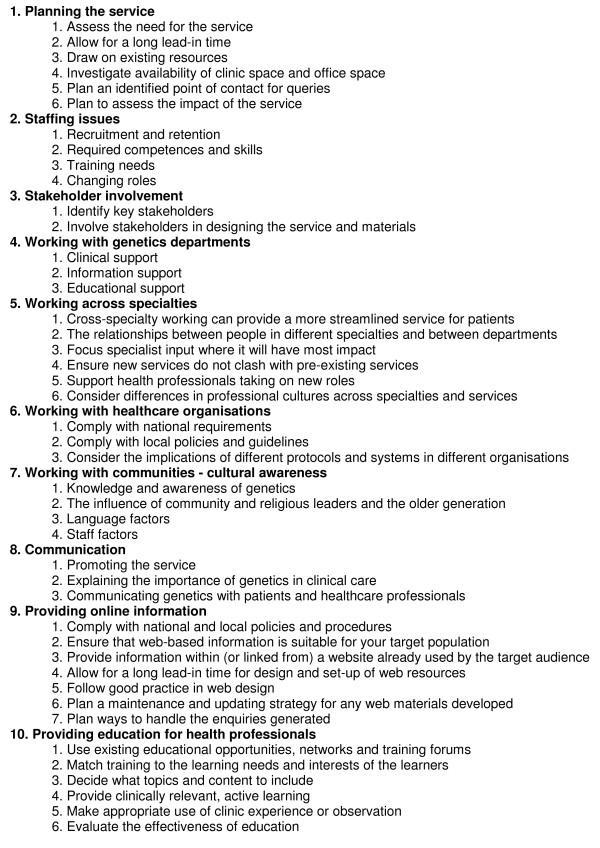
**A Toolkit for developing services for patients with or at risk of genetic conditions**. The full Toolkit with customisable resources is available online at http://www.geneticseducation.nhs.uk.

These themes represent key issues which may need to be considered by clinicians, managers and commissioners when incorporating genetics into mainstream services. The 'practice points' have been collated into an online 'Toolkit', outlined in Figure 2 and available in full from the NHS National Genetics Education and Development Centre's website [16]. The online Toolkit also includes resources from previous service development initiatives, such as job descriptions and clinical tools, which can be customised and reused in new service developments.

## Discussion

Advances in genetic science are being translated into changes in clinical care for patients. More genetic tests are becoming available for disorders in many specialties. For example, more than 50 inherited cardiovascular conditions have now been recognised and genetic tests are available for some of the more common (e.g. familial hypercholesterolaemia) as well as some of the rarer conditions [17]. The organisation and delivery of health services is also changing more generally. For example, in England, the NHS plan outlined a "vision of a health service designed around the patient" [18] and the 'Darzi Report' described how the challenges of the 21^st ^century, such as, "rising expectations; demand driven by demographics; the continuing development of our 'information society'; advances in treatments; the changing nature of disease; and changing expectations of the health workplace", would be met [19]. Thus the development of new roles and services incorporating genetics is occurring in the context of change and modernisation of health services as well as advances in genetics.

Specialist genetics services work closely with a number of specialties to provide co-ordinated patient care and initiatives such as joint clinics have been established (see for example [20-22]). New opportunities for patient care arising from developments in genetics mean that service developments are bringing together different provider organisations, professions and specialties, to develop new roles and services which cross traditional professional boundaries and cultures. This raises a number of challenges which were faced by the 'Mainstreaming Genetics' pilots in planning and developing their new services. Issues of 'ownership' included commissioning and funding of services as well as management of services and staff and physical location, acceptance by staff from other specialties and the importance of formal backing of all stakeholders, including the genetics department.

The development of new roles for health professionals in response to the changing needs of patients is not unique to integrating genetics. For example, General Practitioners in England are being encouraged to develop expertise of a more specialist nature through the development of General Practitioner with a Special Interest roles (GPwSIs) [18]. GPs developing specialist roles face a number of challenges, including workload issues, the need for trust from colleagues and patients and good links with the relevant secondary care specialty [23]. The experiences of the 'Mainstreaming Genetics' pilots indicate similar challenges: in gaining and integrating specialist knowledge from two clinical areas, in developing new career pathways, and in establishing integrated and accepted services. As the pilots experienced, the challenges of establishing new roles must be overcome for the development of services to be successful.

The pilots demonstrated the need to consider the necessary genetics skills and training of staff at an early planning stage, as well as throughout the life of the service. A low level of knowledge of genetics - in society and health professionals - was identified as a key barrier to success. Providing clear information about the service, its aims and benefits, supported by targeted education where appropriate, were found to be essential to gaining buy-in from senior colleagues in organisations as well as from patients and referring health professionals. Competences in genetics for health professionals who are not genetics specialists have since been developed in the UK by Skills for Health working with the NHS National Genetics Education and Development Centre and can be used in defining roles and developing services, and in determining and meeting educational needs [24]. The need to raise awareness of genetic conditions and services within communities in order to improve access to genetics services is an issue which has been highlighted internationally (see for example [25] and [26]).

Some issues were raised by the familial implications of genetic conditions. Within most specialties the focus of care is on the treatment of an individual presenting patient. However, within the specialty of genetics there can be a need to consider the wider family [27]. When services are developed for heritable conditions in other specialties, this 'familial' emphasis can demand a shift in thinking for health professionals. The pilots also identified important 'systems' implications of a 'genetic approach', for example, data systems were sometimes unable to save family history information or to link members of a family. Issues of confidentiality and consent for sharing family information [28] were also raised.

## Conclusions

As our understanding of the role of genetic factors in health and disease increases, new services, new roles and new ways of working need to be developed to translate these advances into benefits for patients. The key themes presented here represent the combined experiences from a range of genetics service developments, in different areas of mainstream medicine, for different genetic conditions and implementing different service models. Many of the new roles and services developed crossed traditional specialty boundaries and brought together different professional cultures. In establishing new services, the pilots defined and tackled key questions for health services: what genetic activities are needed for patient care, who will carry out these genetic activities, how will services be organised and what training, education and support are needed for staff in new roles and delivering new services? They identified some important factors for successful integration of genetic activities within other mainstream medical specialties: working with specialist genetics services to plan and provide clinical and educational support for new roles; understanding the working practices of all specialties involved; identifying key stakeholders, including senior management, relevant health professionals and patients, and involving them at an early stage; demonstrating the importance of genetics and sometimes improving the genetic literacy of health professionals, patients or the public in order to improve use of services.

Providers of healthcare will need to continue responding to advances in genetics as applications to patient care increase. It is important that the experiences from previous genetics service developments are distilled and recorded to prevent the need to 'reinvent the wheel'. Consideration of the issues outlined here will help ensure that the next generation of service developments incorporating genetics into mainstream medical services builds on this body of experience, facilitating the development of high quality services for patients with or at risk of genetic conditions.

## List of abbreviations used

DH: Department of Health for England; GC: Genetic Counsellor; GP: General Practitioner; GPwSI: General Practitioner with a Special Interest; NHS: National Health Service; NICE: National Institute for Health and Clinical Excellence; PCT: Primary Care Trust; RGC: Regional Genetics Centre; UKGTN: United Kingdom Genetics Testing Network.

## Competing interests

The authors declare that they have no competing interests.

## Authors' contributions

PF and HB conceived of the study, participated in its design and were involved in revising the manuscript. CB participated in the study design, data collection, analysis, interpretation of data and was involved in drafting the manuscript. SB participated in data collection, analysis, interpretation of data and was involved in drafting the manuscript. All authors read and approved the final manuscript.

## Pre-publication history

The pre-publication history for this paper can be accessed here:

http://www.biomedcentral.com/1472-6963/10/125/prepub
